# Transforming growth factor-β1-induced N-cadherin drives cell–cell communication through connexin43 in osteoblast lineage

**DOI:** 10.1038/s41368-021-00119-3

**Published:** 2021-04-13

**Authors:** Yueyi Yang, Wenjing Liu, JieYa Wei, Yujia Cui, Demao Zhang, Jing Xie

**Affiliations:** State Key Laboratory of Oral Diseases & National Clinical Research Center for Oral Diseases & Chinese Academy of Medical Sciences Research Unit of Oral Carcinogenesis and Management & West China Hospital of Stomatology, Sichuan University, Chengdu, China

**Keywords:** Adherens junctions, Immunochemistry

## Abstract

Gap junction (GJ) has been indicated to have an intimate correlation with adhesion junction. However, the direct interaction between them partially remains elusive. In the current study, we aimed to elucidate the role of N-cadherin, one of the core components in adhesion junction, in mediating connexin 43, one of the functional constituents in gap junction, via transforming growth factor-β1(TGF-β1) induction in osteoblasts. We first elucidated the expressions of N-cadherin induced by TGF-β1 and also confirmed the upregulation of Cx43, and the enhancement of functional gap junctional intercellular communication (GJIC) triggered by TGF-β1 in both primary osteoblasts and MC3T3 cell line. Colocalization analysis and Co-IP experimentation showed that N-cadherin interacts with Cx43 at the site of cell–cell contact. Knockdown of N-cadherin by siRNA interference decreased the Cx43 expression and abolished the promoting effect of TGF-β1 on Cx43. Functional GJICs in living primary osteoblasts and MC3T3 cell line were also reduced. TGF-β1-induced increase in N-cadherin and Cx43 was via Smad3 activation, whereas knockdown of Smad3 signaling by using siRNA decreased the expressions of both N-cadherin and Cx43. Overall, these data indicate the direct interactions between N-cadherin and Cx43, and reveal the intervention of adhesion junction in functional gap junction in living osteoblasts.

## Introduction

Cell–cell communication, a characteristic feature of virtually all multicellular organisms, mediates a series of responses to the internal or external environment necessary for cell survival. Typically, cell–cell communication occurs between cell surfaces is assured by specialized intercellular protein channels, such as gap junctions or myoendothelial junctions.^1^ Gap junctions (GJs) is critical for coordinating the functions of cells by allowing direct diffusion of ions and small molecules smaller than 1.2 kD (including second messengers like Ca^+^, inositol triphosphate (IP_3_), cyclic nucleotides, and oligonucleotides).^2^ Cell–cell communication mediated by gap junctions is actively involved in virtually all aspects of cellular life cycle, ranging from cell growth, differentiation, and function to cell death. The cell–cell communication in osteoblast lineage can modulate the transcriptional activity of osteoblast-specific promoters, and influence their ability to differentiate and form mineralized matrix during osteoblastic differentiation.^3^ In vertebrates, gap junctions are formed by members of a family of proteins that are called connexins (Cxs). Six identical or different connexin proteins give rise to a hexameric structure called hemichannel (connexon), and a conjunct of the two hemichannels of adjacent cells lead to the formation of a complete channel, the gap junction.^4^ Of all 20 murine and 21 human connexins, connexin 43 (Cx43) is reported to be a major GJ protein. It has been reported that many factors are involved in regulating Cx43, such as vascular endothelial growth factor (VEGF)^5^ and transforming growth factor-β1 (TGF-β1).^6,7^ However, TGF-β has been shown to regulate Cx43 expression in a cell type-dependent manner and the effect of TGF on Cx43 in osteoblast is still unknown.^8,9^ The diverse and physiologically important functions of Cx43 are drawing tremendous attention.

Recent evidence suggests that adherens junction formation is a prerequisite for gap junction assembly. Previous data indicated that potential role of N-cadherin in Cx43 visa interacting with microtubule plus-end-tracking proteins.^10,11^ N-cadherin belongs to a large superfamily with more than 100 types of cadherins and is the core components of adherens junctions. The extracellular domains of N-cadherin on opposing cells bind with each other via a homotypic, transinteraction, leading to the formation of cell–cell adhesions in multicellular organisms.^12^ In recent years, Cx43 protein has been implicated in the trafficking of cardiac ion channels, such as Nav1.5 and junctional protein neural cadherin.^2^ A significant decrease in the gap junction proteins, connexin-43 and connexin-40, was observed in the cadherin-depleted myocytes.^13^ This was first indicated by studies in which disruption of cadherin-containing cell adhesion junctions (AJs) were shown to block gap junction formation.^14,15^ When the N-cadherin gene is deleted in the N-cadherin knockout mice, cardiomyocytes are Cx43 gap junction-deficient.^16^ It is also reported that Cx43 associated with an N-cadherin-containing multiprotein complex is required for gap junction formation in NIH3T3 cells.^17^ However, some converse data have been reported regarding the role of N-cadherin in the formation of gap junction. For example, Govindarajan et al.^18^ reported that, in rat liver epithelial cells, the expression of N-cadherin attenuated GJ assembly by causing endocytosis of Cx43.

Based on these contradictories, we aimed to explore the effect of N-cadherin on TGF-β1-mediated cell–cell communication in osteoblast lineage and its mechanism. We hypothesize that N-cadherin is a key factor in the regulation of gap junctional intercellular communication (GJIC). In the current study, we found that TGF-β1 promote the expression of both Cx43 and N-cadherin. Co-immunoprecipitation indicated coassemble of Cx43 and N-cadherin into a multiprotein complex. This was further confirmed by double-labeled immunofluorescence staining, which showing colocalization of these two proteins at the cell–cell contact sites. Incubation of TGF-β1 enhanced this colocalization. We also found that knockdown of N-cadherin was not only GJIC reduced, but also the effect of TGF-β1 on GJIC was abolished. In addition to affecting partner protein of Cx43, Smad signaling was also involved in the role of N-cadherin in TGF-β1-mediated cell–cell communication.

## Results

### TGF-β1 increases the expression of N-cadherin in osteoblast lineage

**Fig. 1:**
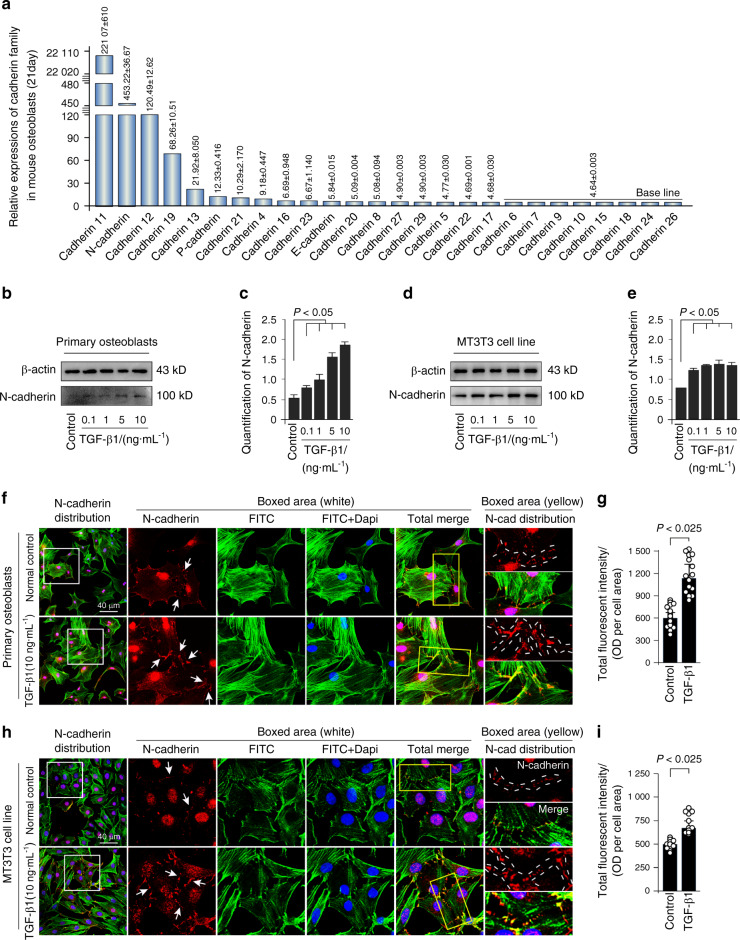
TGF-β1 promotes expression of N-cadherin in primary osteoblasts and MC3T3 cell line. **a** Gene expression of cadherin family in osteoblasts. RNA sequencing showing high expression of N-cadherin among cadherin family in osteoblasts from 21-day-old mice. **b**, **c** Western blotting showing the protein expression of N-cadherin in primary osteoblasts after treatment of TGF-β1 for 48 h. The results were based on three independent experiments (*n* = 3). Quantification in **c** was performed to confirm the protein changes in **b**. *P < 0.05. **d**, **e** Western blotting showing the protein expression of N-cadherin in MC3T3-E1 cells after treatment of TGF-β1 for 48 h. The results were based on three independent experiments (*n* = 3). Quantification in **e** was performed to confirm the protein changes in **d**. *P < 0.05. **f**, **g** Representative IF staining by CLSM showing the increased expression of N-cadherin in primary osteoblasts in response to TGF-β1 (10 ng·mL^−1^). (Cytoskeleton, green; N-cadherin, red; Nucleus, blue). The results were based on three independent experiments (*n* = 3). Quantification in **g** was performed to confirm fluorescent intensity of N-cadherin in **f**. *P < 0.025. **h**, **i** Representative IF staining by CLSM showing the increased expression of N-cadherin in MC3T3-E1 cells in response to TGF-β1 (10 ng·mL^−1^). (Cytoskeleton, green; N-cadherin, red; Nucleus, blue). The results were based on three independent experiments (*n* = 3). Quantification in **i** was performed to confirm fluorescent intensity of N-cadherin in **h**, *P < 0.025

We first showed the expression of cadherin family, which is responsible for adherens junction formation, in osteoblasts isolated from 21-day-old mice by RNA sequencing in our lab (Fig. 1a). The results showed that the expression of N-cadherin ranked the second high among the cadherin members in osteoblasts. To examine the effect of TGF-β1 on N-cadherin protein expression, both primary osteoblasts and MC3T3-E1 cells were incubated with different concentration of TGF-β1 (0.1, 1, 5, and 10 ng·mL^−1^), and we found that TGF-β1 regulate N-cadherin in a dose-dependent manner (Fig. 1b, d). The expression of N-cadherin was approximately 4-fold and 2-fold higher induced by 10 ng·mL^−1^ TGF-β1 in primary osteoblast and MC3T3-E1 cells, respectively (Fig. 1c, e). To explore the distribution of N-cadherin in osteoblasts induced by TGF-β1, we performed immunofluorescence staining (Fig. 1f, h). It was found that N-cadherin mainly localized at cell–cell contact sites, and presented a weak cytoplasmic expression in both primary osteoblasts and MC3T3-E1 cells. After TGF-β1 (10 ng·mL^−1^) treatment, the expression of N-cadherin was largely enhanced (details could be seen in the boxed area). Fluorescent signal of N-cadherin was almost up to 1.50-fold and 1.25-fold to the control groups in primary osteoblasts and MC3T3 cell line, respectively (Fig. 1g, i).

### TGF-β1 upregulates Cx43 expression and promotes gap junctional intercellular communication in osteoblast lineage

**Fig. 2:**
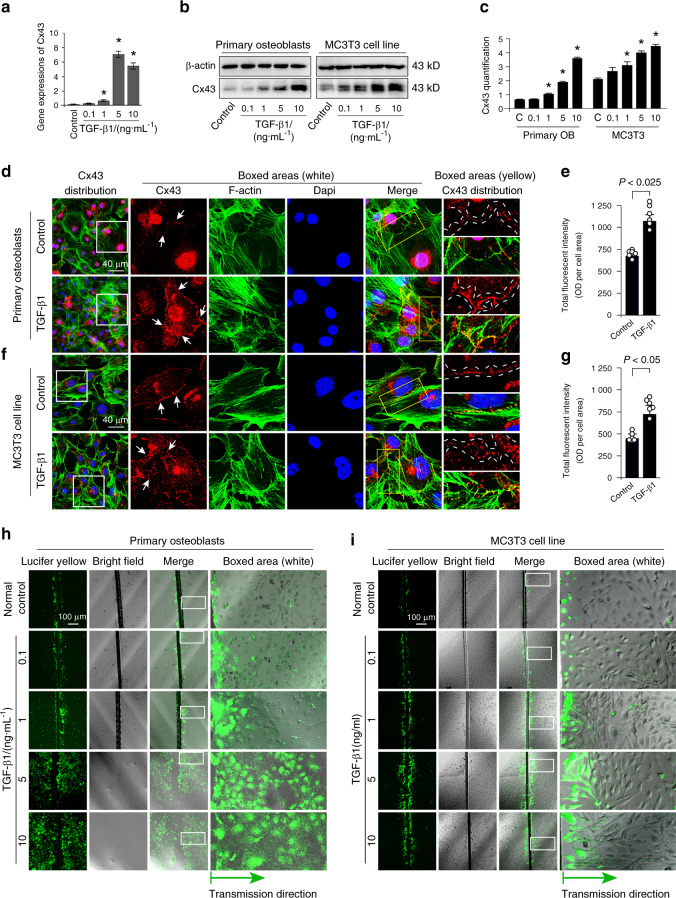
TGF-β1 increases Cx43 expression and promotes the activity of gap junctions in primary osteoblasts and MC3T3 cell line. **a** q-PCR showing gene expression of Cx43 in primary osteoblasts after incubation of TGF-β1 for 24 h. The results were based on three independent experiments (*n* = 3), *P < 0.05. **b**, **c** Western blotting showing the protein expressions of Cx43 in primary osteoblasts and MC3T3-E1 cells in response to TGF-β1 for 48 h. The results were based on three independent experiments (*n* = 3). Quantification in C was performed to confirm the protein changes in **b**. *P < 0.05. **d**, **e** Representative IF staining by CLSM showing the increased expressions of Cx43 in primary osteoblasts in response to TGF-β1 (Cytoskeleton, green; Cx43, red; Nucleus, blue). The results were based on three independent experiments (*n* = 3). White arrows shows the distribution of Cx43 was at the sites of membrane contact. Details are magnified in the boxed areas (yellow). Quantitative analysis of fluorescent intensity of Cx43 in **e** was performed to confirm the protein changes in **d**, *P < 0.025. **b**, **g** Representative IF staining by CLSM showing the increased expressions of Cx43 in MC3T3-E1 cells in response to TGF-β1 (Cytoskeleton, green; Cx43, red; Nucleus, blue). The results were based on three independent experiments (*n* = 3). White arrows shows the distribution of Cx43 was at the sites of membrane contact. Details are magnified in the boxed areas (yellow). Quantitative analysis of fluorescent intensity of Cx43 in **g** was performed to confirm the protein changes in **f**, *P < 0.025. **h**, **i**. The scrape loading/dye transfer (SL/DT) assay showing the changes of dye transfer speed which represents functional GJIC in living primary osteoblasts and MC3T3-E1 cells induced by different concentration of TGF-β1. The images were collected at 7 min after Lucifer yellow stain in living cells along the scrape. The results were based on three independent experiments (*n* = 3)

The expression of Cx43, one of the ubiquitous channel forming proteins, in osteoblasts isolated from 2-day-old mice was tested by qRCR after administration of different concentrations of TGF-β1. As shown in Fig. 2a, treatment with 1, 5, and 10 ng·mL^−1^ TGF-β1 significantly upregulated Cx43 gene expression in primary osteoblasts. We next confirmed the protein levels of Cx43 in osteoblast lineage after TGF-β1 treatment at 48 h. The Western blotting results indicated that Cx43 expression was significantly upregulated by 1, 5 and 10 ng·mL^−1^ TGF-β1 (Fig. 2b, c). To further explore the distribution of Cx43 in osteoblasts induced by TGF-β1, we performed immunofluorescences and found that Cx43 mainly located in the cytoplasm and on cell membrane. Cx43 displayed a punctate-like distribution and even clustered as plaque at the sites of membrane contact between adjacent cells (Fig. 2d, f). Total fluorescent intensity of Cx43 induced by TGF-β1 was more than 1.5-fold that of the control group (Fig. 2e, g). To test the changes of functional GJIC, cells were scrape-loaded with Lucifer yellow and dye transfer was assessed. After 7 min, osteoblasts treated with TGF-β1 showed more avid Lucifer yellow transfer speed extending to the peripheral cells from the wound edge compared with the control group. The enhanced GJIC activity was most significant in the 5 and 10 ng·mL^−1^ TGF-β1 incubated groups in both primary osteoblast and MC3T3-E1 cells (Fig. 2h, i).

### N-cadherin colocalizes with Cx43 to form a protein complex in osteoblast lineage

**Fig. 3:**
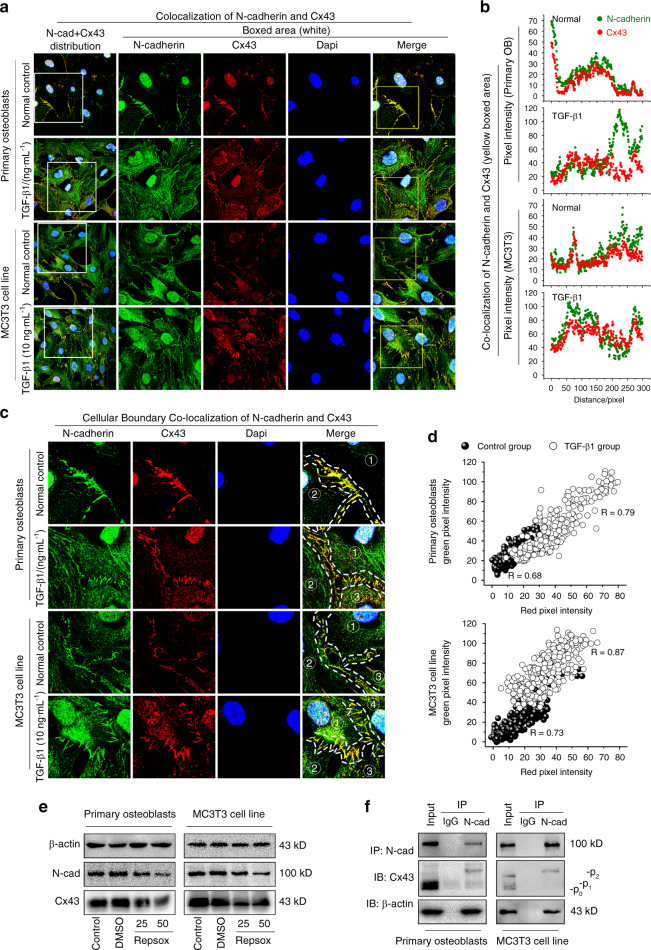
TGF-β1 promotes the interaction between Cx43 and N-cadherin. **a** Double immunofluorescent labeling showing colocalization of Cx43 and N-cadherin in primary osteoblasts and MC3T3-E1 cells. The green signal represents N-cadherin and red signal represents Cx43. The results were based on three independent experiments (*n* = 3). **b** Intensity traces by Image J showing the colocalization distributions of N-cadherin and Cx43 in boxed yellow areas in **a**. **c** Cellular boundary colocalization of N-cadherin and Cx43 in primary osteoblasts and MC3T3-E1 cells. Dotted lines indicate cellular boundary colocalization. ①, ②, ③, and ④ marked adjacent cells. The colocalization area presented orange, which was the overlay of green and red. **d** The Pearson’s correlation of N-cadherin and Cx43 in cellular boundary colocalization is shown in scatter grams in primary osteoblasts and MC3T3 cells based on three independent experiments (*n* = 3). **e** Western blotting showing the expression of N-cadherin and Cx43 in primary osteoblasts and MC3T3-E1 cells after treatment Repsox (TGF-β1 inhibitor) for 48 h. The results were based on three independent experiments (*n* = 3). **f** Co-IP assay further confirmed the interaction between Cx43 with N-cadherin in primary osteoblasts and MC3T3-E1 cells. The result indicates Cx43 is one of the N-cadherin immunoprecipitates. The results were based on three independent experiments (*n* = 3)

We carried out double immunolabeling with antibodies to Cx43 and N-cadherin and found colocalization of the two proteins at the sites of cell–cell contact (Fig. 3a). Details could be seen in the boxed area. Corresponding fluorescence intensity traces of Cx43 and N-cadherin induced by TGF-β1 in the boxed area were plotted (Fig. 3b). To further observe the colocalization of Cx43 and N-cadherin, we magnified the cellular boundary (Fig. 3c). It can be clearly seen that both Cx43 and N-cadherin punctually localized in the contact area of two adjacent cells. Colocalization of the fluorescence signals was on the diagonal area of the scatterplots (Fig. 3d). A more quantitative assessment of colocalization was performed using colocalization coefficients calculated for pixel values contained within the region of interest: Pearson’s correlations (*Rp*). As for primary osteoblasts, the Pearson’ correlation coefficients of Cx43 and N-cadherin colocalization are 0.68 in the control group and 0.79 in the TGF-β1 group, both belong to the “strong” level according to Vadim’s research.^19^ As for MC3T3-E1 cells, Pearson’ correlation coefficient are 0.73 in the control group and 0.87 in the TGF-β1 group, both belong to the “very strong” level according to Vadim’s research. We then performed an inhibition experimentation of TGF-β1 by using Repsox, a potent and specific inhibitor of TGF-β type I receptor.^20^ As shown in Fig. 3e, treatment with 25 and 50 μM Repsox downregulated N-cadherin and Cx43 protein levels in primary osteoblasts and MC3T3-E1 cells. Finally, Co-IP assay was performed to further confirm the interactions between N-cadherin and Cx43 (Fig. 3f). Immunoprecipitations with N-cadherin antibody followed by Western blotting with a Cx43 antibody yielded a predominant p_2_ band of Cx43, one of the phosphorylation forms of Cx43, in both primary osteoblast and MC3T3-E1 cells. Interestingly, β-actin was also yielded by N-cadherin in both cells.

### TGF-β1-induced cell–cell communication through Cx43 requires N-cadherin

**Fig. 4:**
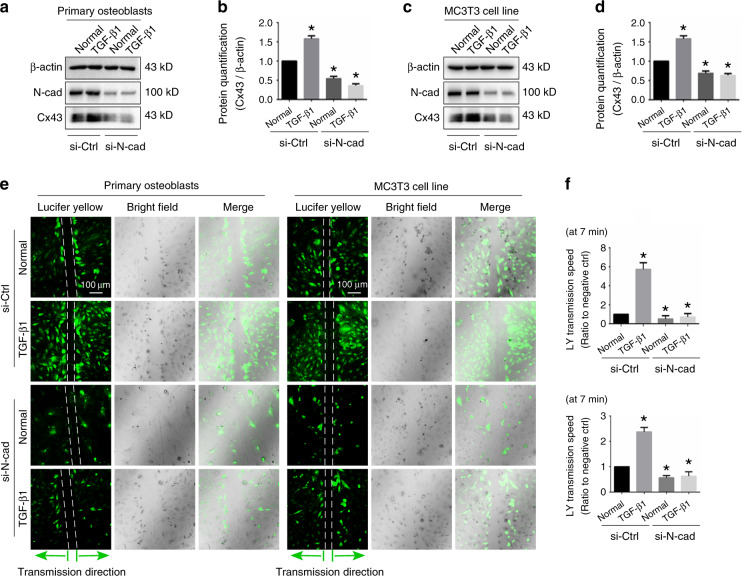
N-cadherin is requisite for TGF-β1-induced functional GJIC. **a**, **b** Western blotting showing the expression of N-cadherin and Cx43 in primary osteoblasts treated with TGF-β1 (10 ng·mL^−1^) in the presence (+) or absence (−) of 100 nmol·L^−1^ N-cadherin siRNA. The results were based on three independent experiments (*n* = 3). Quantification in **b** was performed to confirm the protein changes in **a** (*n* = 3). *P < 0.05. **c**, **d** Western blotting showing the expression of N-cadherin and Cx43 in MC3T3-E1 cells treated with TGF-β1 (10 ng·mL^−1^) in the presence (+) or absence (−) of 100 nmol·L^−1^ N-cadherin siRNA. The results were based on three independent experiments (*n* = 3). Quantification in **d** was performed to confirm the protein changes in **c** (*n* = 3). *P < 0.05. **e** The scrape loading/dye transfer (SL/DT) assay showing functional GJIC in living primary osteoblasts and MC3T3-E1 cells treated with TGF-β1 (10 ng·mL^−1^) in the presence (+) or absence (−) of 100 nmol·L^−1^ N-cadherin siRNA. The images were collected at 7 min after Lucifer yellow staining along the scrape. The results were based on three independent experiments (*n* = 3). **f** Statistical analysis showing the different transmission speed of Lucifer yellow in cells treated with TGF-β1 (10 ng·mL^−1^) in the presence (+) or absence (−) of 100 nmol·L^−1^ N-cadherin siRNA. Upper histogram indicates the changes in primary osteoblasts and lower histogram indicates the changes in MC3T3-E1 cells. Data are presented as mean ± SEM (*n* = 3), *P < 0.05

To determine the role of N-cadherin for the TGF-β1-induced mediation of Cx43, primary osteoblast and MC3T3-E1 cells were treated with TGF-β1 (10 ng·mL^−1^) in the presence or absence of 100 nmol·L^−1^ N-cadherin siRNA. Western blotting showed that expression of N-cadherin was effectively knocked-down in both control and TGF-β1 treated groups. More importantly, pretreatment with 100 nmol·L^−1^ N-cadherin siRNA abolished the stimulatory effect of TGF-β1 on Cx43 protein level (Fig. 4a–d). To investigate whether N-cadherin is essential for TGF-β1-induced GJIC activity, we performed scrape loading/dye transfer (SL/DT) assay (Fig. 4e). Consistent with the western blotting, treatment with TGF-β1 increased GJIC activity, with the transmission speed of LY dye increased, whereas knock-down of N-cadherin attenuated the GJIC activity and also abolished TGF-β1-induced up-regulation of GJIC (Fig. 4f). The combined results suggest that N-cadherin was requisite for TGF-β1-induced upregulation of GJIC mediated by Cx43.

### Smad3/4 signaling is involved in the role of N-cadherin for TGF-β1-induced Cx43

**Fig. 5:**
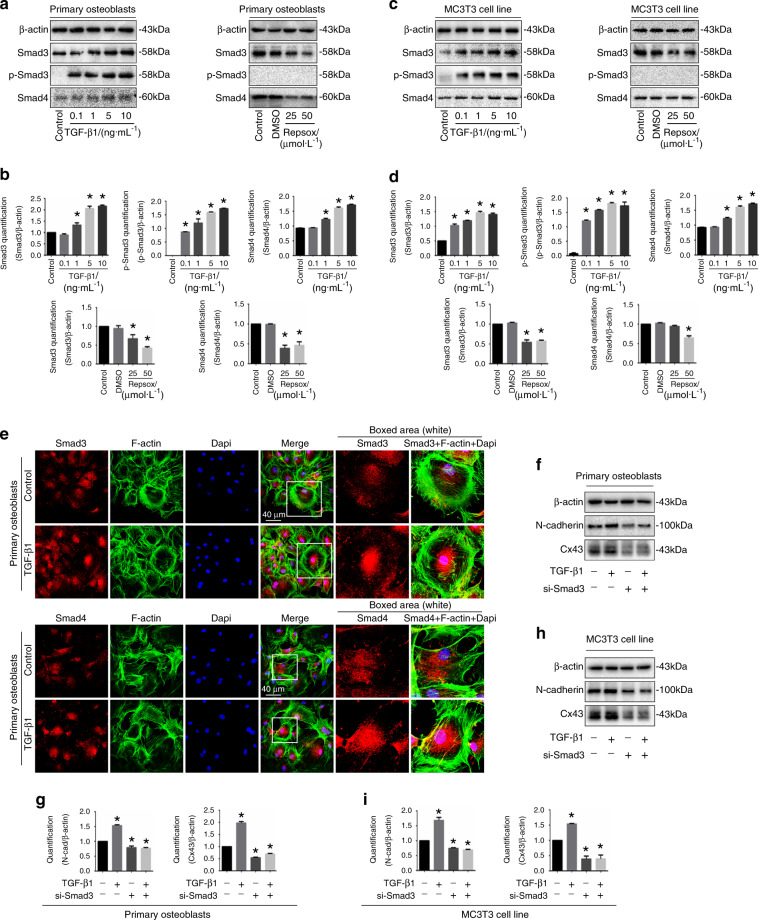
Smad3/4 signaling pathway is involved in the interaction between N-cadherin and Cx43 induced by TGF-β1. **a** Western blotting showing the expression of p-Samd3, Smad3, and Smad4 of primary osteoblast after incubaiton of TGF-β1 and Repsox, respectively. The results were based on three independent experiments (*n* = 3). **b** Quantifications were performed to confirm the protein changes of **a** (*n* = 3). *P < 0.05. **c** Western blotting showing the expression of p-Samd3, Smad3 and Smad4 of MC3T3-E1 cells after incubation of TGF-β1 and Repsox, respectively. The results were based on three independent experiments (*n* = 3). **d** Quantifications were performed to confirm the protein changes of **c** (*n* = 3). *P < 0.05. **e** Representative IF staining by CLSM showing the nuclear accumulation of Smad3 (upper) and Smad4 (lower) in primary osteoblasts in response to TGF-β1 (Cytoskeleton, green; Samd3 and Smad4, red; Nucleus, blue). The results were based on the three independent experiments (*n* = 3). **f** Western blotting showing the protein changes of N-cadherin and Cx43 in primary osteoblasts treated with TGF-β1 (10 ng·mL^−1^) in the presence (+) or absence (−) of 100 nmol·L^−1^ Smad3 siRNA (si-Smad3). The results were based on the three independent experiments (*n* = 3). **g** Quantification was performed to confirm the protein changes in **f** (*n* = 3). *P < 0.05. **h** Western blotting showing the protein changes of N-cadherin and Cx43 in MC3T3-E1 cells treated with TGF-β1 (10 ng·mL^−1^) in the presence (+) or absence (−) of 100 nmol·L^−1^ Smad3 siRNA (si-Smad3). The results were based on the three independent experiments (*n* = 3). **i** Quantification was performed to confirm the protein changes in **h** (*n* = 3), *P < 0.05

TGF-β1 regulates cellular functions through activation of the Smad-dependent signaling cascade.^21–23^ Upon TGF-β1 treatment, Smad2 and Smad3 are phosphorylated and attract Smad4 to form complexes. Those complexes then translocate into the nuclei to regulate target gene expression.^24^ In the study, we showed TGF-β1 increased Smad3, p-Smad3 and Smad4 expression and Repsox decreased Smad3/4 expression in primary osteoblasts (Fig. 5a, b) and MC3T3-E1 cells (Fig. 5c, d). By immunofluorescent staining, we found that TGF-β1 induced Smad3 and Smad4 to accumulate into the nuclear region (Fig. 5e). To examine the involvement of Smad3/4 signaling in the TGF-β1-induced upregulation of Cx43 and N-cadherin, we utilized siRNA of Smad3 to block the Smad signaling. As shown in Fig. 5f–i, siRNA of Smad3 downregulated the Cx43 and N-cadherin in both primary osteoblasts and MC3T3-E1 cells. Importantly, knockdown of Smad3 also abolished the TGF-β1-induced upregulation of Cx43 and N-cadherin.

## Discussion

It has been well established that gap junction in vertebrates are comprised with connexins, among which Cx43 is the most ubiquitous and abundant. Six connexin43 form the hemichannel (connexon) and two adjacent connexons of opposing cell membrane to form an intercellular channel. These channels concentrated at cell–cell interfaces to form high-density gap junction plaques. In SDS-PAGE, Cx43 separates into multiple bands typically indicated as *P*_0_, *P*_1_, and *P*_2_.^25^ Among all cell types examined, Cx43 is synthesized as a single, 42 kDa species that is transformed to a species of about 44 kDa, i.e., Cx43-P_1_, and then to one of about 46 kDa (Cx43-P_2_) by adding phosphate onto serine residues.^26^ The mutual modulation in the assembly of gap and adherens junctions, suggests that the expression of the functional proteins maybe coordinated.^15^ In this study, we found that N-cadherin interact with Cx43, especially the Cx43-P_2_, by the observation that Cx43-P_2_ coimmunoprecipitates with N-cadherin. We suspect this is because Cx43-P_2_, the phosphorylated form of the protein, is thought to be the major component of large junctional plaques.^25^ It was reported that in some cells with a severe lack of junctional communication, despite the synthesis of Cx43, neither process it to the P_2_ form nor accumulation of gap junctional communication was observed.^26^ Processing of Cx43 to the P_2_ form is thus required to form complexes with N-cadherin, and is involved in gap junctional plaque assembly or in functional process.

TGF-β signal pathway regulates various developmental, physiological, and pathological processes.^27^ In this study, we found that TGF-β upregulate cell–cell communication through Cx43 in osteoblast. It is well-known that TGF-β exerts its biological effects by activating SMAD-dependent and SMAD-independent pathways. In SMAD-dependent pathway, TGF-β1 bind to TGF-β receptors and then attract the downstream molecule Smad3, which were phosphorylated and attract Smad4 to form complexes, those complexes subsequently translocated into the nuclei to regulate target gene. Our results demostrate that administration of exogenous recombinant TGF-β1 induced the expression of Smad3, p-Smad3 and Smad4 and a translocation of Smad3/4 into nucleus was detected. Moreover, after knocking-down of Smad3, the expression of Cx43 was reduced and the upregulation effect of TGF-β1 on Cx43 was abrogated. Thus TGF-β/Smads signaling pathway is one of the most important pathways that are activated. However, this is not the only pathway which can modulate Cx43 by TGF-β1 since the expression of Cx43 was not abrogated by using siRNA Smad3. We assume that there may be other signal pathway involving in the regulation of Cx43 in osteoblasts. It will be interesting to identify, which pathway is also involved in the process of inducing Cx43 by TGF-β1 in future studies.

Cell-cell interactions are mediated by proteins such as cadherins which ensure cell–cell adhesion, connexins which are involved in cell–cell communications by forming GJs. Cadherins have been thought to facilitate the assembly of connexins (Cxs) into gap junctions (GJs) by enhancing cell-cell contact. Expressed alone in cells, E-cadherin and N-cadherin exhibit different effect on GJs. However, when both cadherins are simultaneously expressed in the same cell type, GJ assembly and disassembly occur concurrently.^18^ In our study, we found that in murine calvaria osteoblasts, N-cadherin was highly expressed while the expression of E-cadherin was low. We speculate that N-cadherin plays a predominant role on the assembling of GJs. This conclusion is based on the results of colocalization and coprecipitation, which demonstrate that in confluent osteoblasts, cadherins interacts with Cx43, a requisite for cell–cell communication. In this study, we found that Cx43 and N-cadherin showed the same trend of increased expression under induction of TGF-β1. In addition, an increase of intracellular localization of Cx43 as discrete vesicular puncta and a decrease of membrane-distributed Cx43 coincided with siRNA-mediated N-cadherin knockdown. This is consistent with the results in other cell types. For example, N-cadherin is predominantly expressed in cardiac myocytes where it is a requisite protein for the assembly of Cx43 into GJs.^28^ In NIH3T3 cells, gap junction formation was shown to involve the aggregation of Cx43 in complexes containing N-cadherin and multiprotein such as α-catenin, β-catenin, p120 and ZO-1.^17^ All these results pointing to interactions between Cx43 and N-cadherin play a vital role in GJs formation. Besides, we also noticed that using lose-of-function of N-cadherin approach could notably eliminate the upregulation of TGF-β1 on Cx43, indicating that TGF-induced Cx43 need N-cadherin. We reason that N-cadherin not only participates in the assembly of Cx43 into GJs, but also maybe involved in the transcription of Cx43. Recently, it is reported that Cx43 is a direct transcriptional regulator of N-cadherin in vivo.^29^ Given the close relationship between Cx43 and N-cadherin, whether and how N-cadherin affect Cx43 transcription remains to be explored. On the other hand, N-cadherin overexpression negatively regulates osteoblast differentiation and function in basal culture conditions. Meanwhile, increased Cx43 expression guarantees the osteoblast differentiation and function. To a certain extent, it also reflects the synergy of cx43 and N-cadherin.^30^ In terms of in vivo experiments, Lai et al. proved that bone mass was not significantly different between N-cadherin (Cdh2)+/− and wildtype littermates, but on ovariectomy, bone loss in Cdh2+/− mice was initially slower, but with time it became significantly greater than in wildtype mice.^31^ Meanwhile, Reaume et al. found that homozygous Cx43−/− pups had swelling in the neck and abdomen at birth. They contracted muscles quickly and died within an hour after giving birth. And heterozygous Cx43−/− mice have delayed intramembranous and intrachondral ossification, which is the result of widespread osteoblast dysfunction.^32^ Recently, Ma et al. showed that Cx43 could protect bone loss during estrogen deficiency.^33^

In summary, our study demonstrated the role of N-cadherin in TGF-β1-mediated cell–cell communication in osteoblast lineage. That TGF-β1 promotes GJIC activity by increasing expression of Cx43 requires the participation of N-cadherin. Nevertheless, we were unable to demonstrate how N-cadherin signals to control the assembly of GJs. Future work about N-cadherin dictates the assembly of GJs could help to understand cell–cell communication.

## Materials and methods

### Primary osteoblast isolation

The protocol for animal experiments was strictly according to ethical principles and approved by our Institutional Review Board (IRB, No.WCHSIRB-D-2017-029). The animal experiments were carried out in the State Key Laboratory of Oral Diseases, West China Hospital of Stomatology, Sichuan University. Primary calvarial osteoblasts were isolated from mouse skulls, as described in previous study.^34^ In brief, 2–3 day new born C57BL/6J mice were obtained from animal center of Sichuan University. The craniums were obtained from 2-day-old to 3-day-old mice anesthetized with sodium pentobarbitone (~1 mg per mouse). After being washed twice in 1× PBS and cut into small pieces, calvarium fragments were trypsinized for 30 min in 0.25% protease solution and replaced by being digested in 1 mg·mL^−1^ type I collagenase (C0130, Sigma-Aldrich, MO, USA) overnight. Then fresh α-MEM (#SH30265; Hyclone, GE Healthcare Life Sciences, Pittsburgh, PA, USA) containing 10% fetal bovine serum (FBS, Invitrogen, Paisley, UK) was added at 1:1 (v/v) to stop digestion. The cells were suspended by centrifuging for 5 min. After the supernatant was removed, tissues and cells were transferred into flasks and cultured under conditions of 95% air and 5% CO_2_ at 37 °C. Osteoblasts at the second passage were used in the current study. Observation of primary osteoblasts under light microscope and morphological staining revealed that the adherent cells were mainly spindle-shaped or triangular, and the circular or elliptical nucleus was in the center or on the side of the cell. The cells have protrusions of varying numbers and lengths, showing a tendency to colonize and grow, and can grow in multiple layers. Primary osteoblasts have different regulation of gene expression at different stages of differentiation.^35^ Alkaline phosphatase and mineralized nodules are markers of osteoblast differentiation.^36^ A large number of cells obtained from the alkaline phosphatase staining experiment showed a positive reaction, and the cell membrane and intracytoplasmic particles were stained as brown or brown particles. Stained with Alizarin Red, orange-red mineralized nodules can be seen. Comprehensive comparison of morphology, alkaline phosphatase staining and mineralization function can be used to identify primary osteoblasts.^37^

### MC3T3-E1 cell culture

Murine MC3T3-E1 (subclone 4) (American Type Culture Collection, Manassas, VA), were cultured in α-MEM medium containing 10% FBS and 1% penicillin–streptomycin solution in a humidified culture chamber under conditions of 95% air and 5% CO_2_ at 37 °C. MC3T3-E1 is a murine pre-osteoblast cell line that can differentiate into osteoblasts when stimulated with BMP-2, which is a well-accepted model for investigating the osteogenic differentiation.^38^

### Quantitative real-time PCR (q-PCR)

Total RNA was isolated with Trizol reagent (Invitrogen) according to the manufacturer’s instructions (cells at 5 × 10^5^ per well (6-well plate) were used as one amount of a sample). RNA from extracted tissues was precipitated with isopropanol and dissolved in DEPC-treated distilled water. cDNA was synthesized by reverse transcription with reverse kits (Thermo scientific, Vilnius, Lithuania). q-PCR was performed with SYBR Premix Ex Taq using iCycler (Bio-Rad). The amplification process was set with 40 cycles to carry out an initial denaturation of 30 s at 94 °C, 20 s annealing at 65 °C, and 10 s extension at 72 °C. Specific primers were designed in Table 1 using Primer Express software (Applied Biosystems). GAPDH was used as an internal control. 2^−ΔΔCt^ method was used to analyze the amount of gene expressions in each group.

**Table 1:** Primer pairs for q-PCR in this study

mRNA	Primer pairs
GAPDH (NM_001289726.1)	Forward: AGGTTGTCTCCTGCGACTTCA
	Reverse: CCAGGAAATGAGCTTGACAAA
Connexin43 (NM_010288.3)	Forward: TGCACCTGGGGTGTTCATTT
	Reverse: GCCGCCTAGCTATCCCAAAA

### Western blotting

The specific procedure in our published paper was followed.^39^ Brief description was as follows: cells at 5 × 10^5^ per well (six-well plate) were lysed as one amount of a sample. Equal amounts of protein extracts were separated on 10% SDS-polyacrylamide gel electrophoresis, and then transferred into a PVDF membrane (Millipore, Germany) at 200 mA for 2 h. PVDF Membranes were blotted with 5% milk for 1 h and then the blots were probed overnight with primary antibodies (β-actin, 1:2 000, sc-47778; Cx43, 1:3 000, #11370; N-cadherin, 1:2000, #76057; Smad3, 1:3 000, #28379; Smad4, 1:5000, #40759; p-Smad3, 1:2 000, #52903; Abcam, Cambridge, UK), then added corresponding secondary antibodies (m-IgG_К_BP-HRP, 1:4 000, sc-516102; mouse antirabbit IgG-HRP, 1:2 000, sc-2357, Santa Cruz) and incubated for 2 h. Signals from PVDF membranes were taken by a Western Blotting Luminol Reagent Kit (Santa Cruz).

### Co-immunoprecipitation (Co-IP)

Protein immunoprecipitation was performed using the Pierce Co-IP Kit (lot no. SB240573B) according to our previously study.^40,41^ Briefly, osteoblasts (5 × 10^6^ cells per sample) were lysed according to the manufacturer, and the antibody of bait protein (N-cadherin) was added into the lysate at a 1:30 ratio. After overnight incubation, the antigen (bait protein) and the interacting proteins (prey proteins) were separated and purified by the centrifugal column provided in the kit. After elution with elution buffer, the samples were collected and blotted by PVDF membrane by Western blotting.

### siRNA interference

For siRNA-mediated knockdown of Smad3 and N-cadherin, small interfering RNAs (Dharmacon), which were prepared and transfected using Lipofectamine RNAiMAX (Invitrogen, Burlington, ON, Canada) according to the manufacture’s protocols to achieve final siRNA concentrations of 100 nmol·L^−1^. The siCONTROL NON-TARGETING *pool* siRNA (Dharmacon) was used as the transfection control (cells at 5 × 10^5^ per well (six-well plate) were used as one amount of a sample). The knockdown efficiency was examined using Western blot analyses. siRNA sequences are available in Table 2 (siRNA SMAD3) and Table 3 (siRNA N-cadherin).

**Table 2:** siRNA SMAD3 sequences in this study

Gene	Strand	Sequence
si-SMAD3	Sense	5′-CAGAACGUGAACACCAAGU-3′
	Antisense	5′-ACUUGGUGUUCACGUUCUG -3′
si-NC	Sense	5′-UUCUCCGAACGUGUCACGU-3′
	Antisense	5′-ACGUGACACGUUCGGAGAA-3′

**Table 3:** siRNA N-cadherin sequences in this study

Gene	Strand	Sequence
si-N-cadherin	Sense	5′-GGACAUCAAUGGCAAUCAA-3′
	Antisense	5′-UUGAUUGCCAUUGAUGUCC-3′
si-NC	Sense	5′-UUCUCCGAACGUGUCACGU-3′
	Antisense	5′-ACGUGACACGUUCGGAGAA-3′

### Immunofluorescence and confocal laser scanning (CLSM)

Immunofluorescence staining was performed as previously described.^42,43^ Briefly, Cells were cultured in Petri dishes specified for confocal laser microscopy for 12 h (1 000 cells per dish, Glass Bottom Cell Culture Dish, Φ15 mm, No. 801002, NEST, Jiangsu, China). Then TGF-β1 (5 ng·mL^−1^, p04202, R&D Systems, USA) were added into the culture media as the experimental group and continued to incubate for 24 h. Then the cells were fixed in 4% paraformaldehyde for 20 min and rinsed with 1× PBS three times. After being penetrated by 0.5% Triton X-100 (Beyotime, Shanghai, China) for 15 min, the samples were blocked with phosphate-buffered saline containing 1% bovine serum albumin (BSA) for 1 h. Cells were then incubated with the antibodies (anti-Cx43, 1:200; N-cadherin, 1:200; Smad3, 1:200; Smad4, 1:200; Abcam, Cambridge, UK) overnight at 4 °C. The secondary antibody was Alexa Fluor® 647 (10 μg·mL^−1^, Alexa Fluor ®647, Life Technology, Grand Island, NY, USA). Nuclei were counterstained with 4′,6-diamidino-2-phenylindole (DAPI; D9542, Sigma, USA) and the cytoskeleton was stained with phalloidin (FITC, A12379, Thermo). Two different antibodies, conjugated with two different fluorochromes (i.e., Alexa Fluor® 488 antibody: green fluorescence; Alexa Fluor® 647 antibody: red fluorescence) were used in double-fluorescence labeling. The immunofluorescence images were observed through a confocal laser scanning microscope (FV3000, Olympus, Tokyo, Japan).

### Scrape loading and dye transfer assay

Scrape loading and dye transfer assay was performed to detect the gap junction intercellular transference in the living cells as previously described.^21,23,44^ Briefly, small molecular (MW < 900) dyes (Lucifer yellow, MW457, L0259, Sigma) were introduced and their intercellular movement through gap junctions was detected by CLSM. Firstly, the adherent cells were treated with TGF-β1 as experimental group. After treatment, cells are required to grow to a full confluence (100%), and then were washed with CaMg-PBS and then three scrapes were made using a surgical blade needle in the presence of 1 mg·mL^−1^ Lucifer yellow (L0259, Sigma). Cells were incubated for 7 min at room temperature. After rinsing thoroughly to eliminate background, dye transfer was captured using CLSM.

### Quantification and statistical analysis

All protein bands were quantified using optical density (OD) value by ImageJ software (ImageJ2, NIH, Bethesda, MD, USA). The statistical analysis was described in our previously published paper.^21,23^ The analyzed results were all presented as mean ± SEM. In each analysis, critical significance level was set to be *P* < 0.05. Pearson’s correlation coefficient (*R*_p_) calculated according to the previous report.^45^

## Data Availability

No publicly available data or shared data are cited. All original data supporting the finding of the current study are available from the corresponding author, Dr. J.X., on request.
